# Asparagine synthetase regulates lung-cancer metastasis by stabilizing the β-catenin complex and modulating mitochondrial response

**DOI:** 10.1038/s41419-022-05015-0

**Published:** 2022-06-23

**Authors:** Dong-Jing Cai, Zi-Yu Zhang, Yue Bu, Li Li, Yue-Zhen Deng, Lun-Quan Sun, Cheng-Ping Hu, Min Li

**Affiliations:** 1 Department of Respiratory Medicine, National Key Clinical Specialty, Branch of National Clinical Research Center for Respiratory Disease, Xiangya Hospital, Central South University, Changsha, China; 2 Xiangya Lung Cancer Center, Xiangya Hospital, Central South University, Changsha, China; 3 Department of Neurology, Xiangya Hospital, Central South University, Changsha, China; 4 Xiangya Cancer Center, Xiangya Hospital, Central South University, Changsha, China; 5 Key Laboratory of Molecular Radiation Oncology of Hunan Province, Changsha, Hunan China; 6 National Clinical Research Center for Geriatric Disorders, Changsha, China; 7 Center of Respiratory Medicine, Xiangya Hospital, Central South University, Changsha, Hunan China; 8 Clinical Research Center for Respiratory Diseases in Hunan Province, Changsha, China; 9 Hunan Engineering Research Center for Intelligent Diagnosis and Treatment of Respiratory Disease, Changsha, Hunan China

**Keywords:** Non-small-cell lung cancer, Cell invasion, Hydrolases

## Abstract

The availability of asparagine is the limitation of cell growth and metastasis. Asparagine synthetase (ASNS) was an essential enzyme for endogenous asparagine products. In our study, ASNS-induced asparagine products were essential to maintain tumor growth and colony formations in vitro. But mutated ASNS which defected endogenous asparagine products still upregulated cell invasiveness, which indicated that ASNS promoted invasiveness by alternative pathways. Mechanically, ASNS modulated Wnt signal transduction by promoting GSK3β phosphorylation on ser9 and stabilizing the β-catenin complex, as result, ASNS could promote more β-catenin translocation into nucleus independent of endogenous asparagine. At the same time, ASNS modulated mitochondrial response to Wnt stimuli with increased mitochondrial potential and membrane fusion. In summary, ASNS promoted metastasis depending on Wnt pathway and mitochondrial functions even without endogenous asparagine products.

## Abstract (web summary)

In the Wnt pathway, phosphorylation of GSK3β(S9) is very important to stabilize β-catenin complex, ASNS can upregulate phosphorylation of AKT on Ser473, then promote GSK3β phosphorylation on Ser9 to stabilize β-catenin complex. When Wnt pathway is activated, ASNS elevates p-DRP1(637) and decreases p-DRP1(616) to suppress mitochondrial fission.

## Graphical abstract

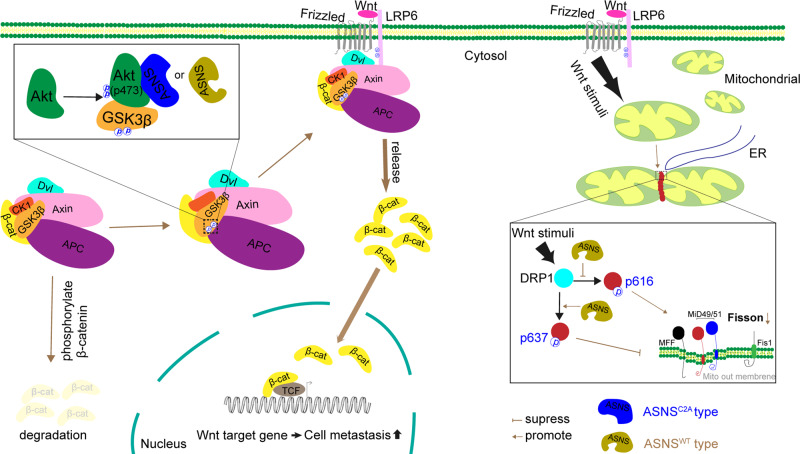

## Introduction

Lung cancer causes millions of deaths each year globally, while metastasis is currently the biggest impediment to the clinical management of lung cancer [1]. Nearly half of lung-cancer patients suffer metastasis after surgery, even in patients who have undergone complete resection [2]. Metastasis of lung cancer is a complex and unspecified process. Adaptive stress response during tumor metastasis is related to the balance of survival signals and the reconstruction of local and distant environments, these need to be further elucidated [3].

Changes in metabolism can directly participate in tumor metastasis and growth. Glutamate and aspartate fuel the TCA cycle and glycolysis by providing carbonic intermediates to produce enough ATP for tumor development and metastasis. Asparagine availability is an endogenous metabolic limitation for tumor growth [4]. High asparagine bioavailability directly against glutamine depletion induced tumor cell death [5]. Luo et al. proposed that the bioavailable conversion of glutamine to asparagine promoted tumor cell survival, growth, and metastasis [6].

Human asparagine synthetase (ASNS) which consists of 561 amino acid residues converts the transfer of acylamino group from glutamine to asparate in an ATP-dependent manner [7]. Human ASNS dysfunction is clinically associated with asparagine synthetase deficiency (ASD) and childhood acute lymphoblastic leukemia. In solid tumors, elevated ASNS expression is correlated with poor overall survival of lung cancer (www.proteinatlas.org). ASNS overexpression can rescue cell apoptosis and cycle arrest owing to ATF4 deficiency or nutrition stress [8, 9]. In the mouse breast cancer model, high ASNS expression is positively correlated with the amount of circulating tumor cells and invasive potential. The increased epithelial-to-mesenchymal transition can partially explain how ASNS promotes cell metastasis, but more details still need to be investigated [5].

We found that ASNS was higher in lung-cancer tissues than normal lung tissue using public databases (https://xenabrowser.net/datapages/) and tissue microarray of lung cancer. ASNS level was insignificantly correlated with the age, gender, treatment, histopathological grade among lung-cancer patients but significantly correlated with the lymph node metastasis. Multivariate Cox regression model analysis also indicated that the overall survival was shorter in patients with high ASNS expression than those with low ASNS expression. ASNS level could be a predictor for survival but more potential values were also needed to elucidate.

## Results

### Higher ASNS is correlated with lung-cancer metastasis

**Fig. 1:**
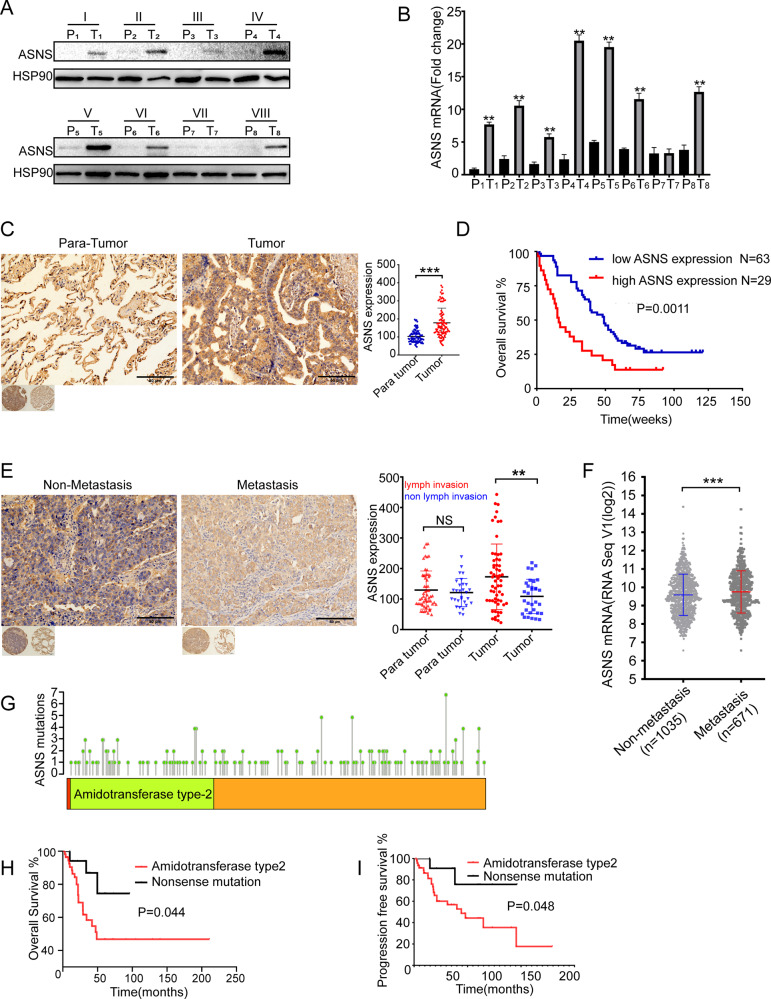
ASNS level is correlated with shorter survival and easier lung-cancer metastasis. Surgical lung-cancer samples were collected and divided into tumor and para-tumor region, P, para tumor, T, tumor, ASNS were detected in protein level (**A**) and mRNA level (**B**). **C** in the tissue microarray of lung cancer, ASNS protein was tested by immunohistochemistry among 92 matched samples and scored by the Quantitative Pathology Imaging System (PerkinElmer), ASNS expressed level was analyzed by ANOVA. **D** 92 patients were divided into high ASNS and low ASNS groups by Immunohistochemical score, and different overall survivals were tested by Log-rank (Mantel–Cox) test. **E** 92 patients were divided into metastasis and non-metastasis groups, the significance of different ASNS levels was tested by Student’s *t* test. **F** 1706 samples were collected from TCGA and grouped by metastasis and non-metastasis, ASNS mRNA level was analyzed by Student’s *t* test, data download from https://xenabrowser.net/datapages/. **G** ASNS protein sequence was annotated, amino acid mutations were also shown on ASNS domains. **H**, **I** survival of different ASNS mutated groups were analyzed by Kaplan–Meier survival analysis. Error bars were expressed as mean ± S.D. *P* < 0.05 is significant statistical difference, *p < 0.05, **p < 0.01, ***p < 0.001. Representative images from three independent experiments are shown.

Eight surgical samples of non-small cell lung cancer (NSCLC) were collected from Xiangya Hospital, then these samples were divided into tumor and para-tumor regions. The tumor region had higher ASNS than the para-tumor region in translational and transcriptional levels (Fig. 1A, B). Then tissue microassay which contained 92 patients’ samples of NSCLC was used. The tumor region had higher ASNS level than the para-tumor region (Fig. 1C). Moreover, patients with higher ASNS had poor overall survival than those with lower ASNS (Fig. 1D). Furthermore, these patients were classified into two groups: metastasis and non-metastasis. The metastasis group had higher ASNS level than the non-metastasis group (Fig. 1E). In The Cancer Genome Atlas Program (TCGA) cohort, 1706 lung-cancer patients were collected, the patients with metastasis had higher ASNS mRNA expression than those without metastasis (Fig. 1F). Multivariate Cox regression analysis showed that clinical stage (HR, 95%CI: 2.08 (1.34,3.22)) and nuclear ASNS expression (HR, 95%CI: 3.84(1.31,11.21)) were high-risk factors for survival (Table 1). In TCGA cohort, 146 patients with ASNS mutations were found, the patients with ASNS mutations in the amidotransferase type-2 domain had poor survival than those with nonsense mutations (Fig. 1G–I). The ASNS would be a strong survival predictor of NSCLC and directly or indirectly participate in cell metastasis.

**Table 1:** The clinical characteristic and *ASNS* level in non-small cell lung-cancer patients (*N* = 92).

Characteristic			COX regression model	
			P*	HR(95%CI)
Age	(30–84 years)		0.781	1.079 (0.63,1.85)
	30–50(9,10%)			
	50–70(61,66%)			
	70–84(23,24%)			
Sex	male(49,53%)		0.524	1.202 (0.682,2.117)
	female(43,47%)			
Pathological degree	I–III		0.527	0.840 (0.491,1.44)
	I(3,3%); II(61,66%);III(28,31%)			
Tumor size	(1.5–14 cm)		0.416	1.183 (0.788,1.776)
	1.5–3.0(18,20%)			
	3.0–4.5(45,49%)			
	≥4.5(29,31%)			
lymph invasion	positive(58,63%)		0.806	0.897 (0.375,2.145)
	negative(34,37%)			
Clinical stages	I–IV		0.001	2.081 (1.343,3.224)
	I(28,30%); II(17,18%);III(47,52,%)			
ASNS expression**	cancer tissue (IHC score)			
	in nuclues	83%low(117 ± 57); 17%high(330 ± 77)	0.014	3.837 (1.313,11.214)
	in cytoplasm	68%low(131 ± 33); 32%high(297 ± 60)	0.065	2.114 (0.954,4.683)
	para-cancer tissue (IHC score)			
	in nuclues	65%low(92 ± 23); 35%high(180 ± 42)	0.686	1.262 (0.41,3.885)
	in cytoplasm	60%low(77 ± 13); 40%high(124 ± 26)	0.237	0.473 (0.137,1.635)

^*^P-value was calculated by COX regression model;

^**^ASNS expression were detected by immunohistochemistry.

### High ASNS promotes invasiveness by alternative pathways beyond asparagine metabolism

**Fig. 2:**
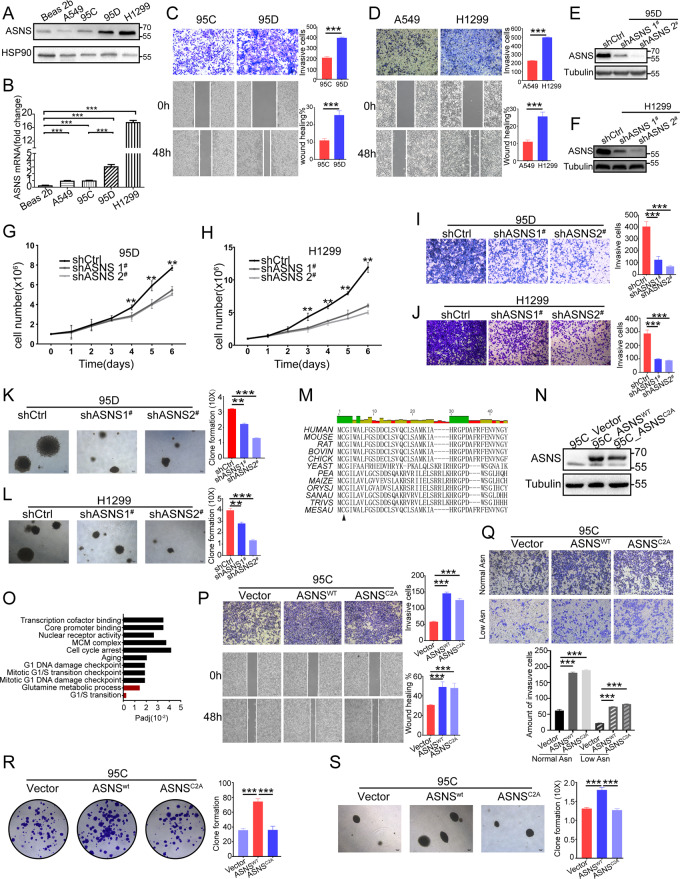
ASNS enhances cellular invasiveness independent of endogenous asparagine products. In different lung-cancer cell lines, ASNS were tested in protein (**A**) and mRNA (**B**) levels, ASNS mRNA level was analyzed by Student’s *t* test. 95C and 95D cell lines were derived from the same lung-cancer patient and domesticated into high metastasis (95D) and low metastasis (95C) cell lines (**C**); H1299 and A549 cell lines were also paired cell lines, their invasive and migrative ability were tested (**D**), Student’s *t* test was used for statistic test. ASNS protein was knocked down in 95D and H1299 (**E**, **F**), cell growth number was recorded (**G**, **H**), and the different growth speeds were tested by Student’s *t* test. Knockdown-ASNS cells were seeded into Matrigel-coated Transwell chambers and cultured in FBS gradient medium for 36 h. The count of invasive cells was recorded in 95D group (**I**) and H1299 group (**J**), the different invasive abilities were tested by Student’s *t* test. At the same time, knockdown-ASNS cells were seeded into colony assay (**K**, **L**), then cultured for 2–3 weeks. **M** aligning ASNS protein sequence in different species. And building ASNS^WT^ and ASNS^C2A^ cell lines (**N**). ASNS^C2A^ cells defected glutamine metabolism and asparagine synthesis according to RNA sequencing data (**O**). The ASNS overexpressed cells were seeded into invasive and migrative assay in normal asparagine medium (**P**) and low asparagine medium (**Q**). At the same time, these cells were seeded into crystal violet staining assay (**R**) and colony formation assay (**S**), then cultured for 2–3 weeks. The clone formations were analyzed by Student’s *t* test. Error bars were expressed as mean ± S.D. *P* < 0.05 is significant statistic difference, *p < 0.05, **p < 0.01, ***p < 0.001. Representative images from three independent experiments are shown.

We firstly detected ASNS level in lung-cancer cell lines: 95C, 95D, A549, H1299, and Beas2b (Fig. 2A, B). 95D (high metastasis) and 95C (low metastasis) was a paired cell line derived from the same patient (Fig. 2C). H1299 and A549 cell line was the other paired cell line. The invasive and migrative abilities were tested (Fig. 2D). The cell lines with higher ASNS levels had stronger invasive and migrative ability than those with lower ASNS levels (Fig. 2C, D). Then we knocked down ASNS in 95D and H1299 (Fig. 2E, F). Knockdown ASNS decreased cell growth (Fig. 2G, H), invasiveness (Fig. 2I, J), and colony formation (Fig. 2K, L). Furthermore, we mutated ASNS protein in the conserved region [10] to defect asparagine product (Fig. 2M, O) and built ASNS mutated cell lines (Fig. 2N). Under normal asparagine or low asparagine condition, overexpressed ASNS^WT^ or ASNS^C2A^ could make cell invasiveness stronger than vector type. Interestingly, we found ASNS^C2A^ had approximately invasive promotion as ASNS^WT^ type (Fig. 2P, Q). At the same time, we could detect a similar invasive promotion in A549-ASNS^C2A^ cell line. Besides, ASNS^C2A^ cells had weaker colony formation than ASNS^WT^ cells (Fig. 2R, S).

### ASNS modulates mitochondrial response to wnt3a stimuli

**Fig. 3:**
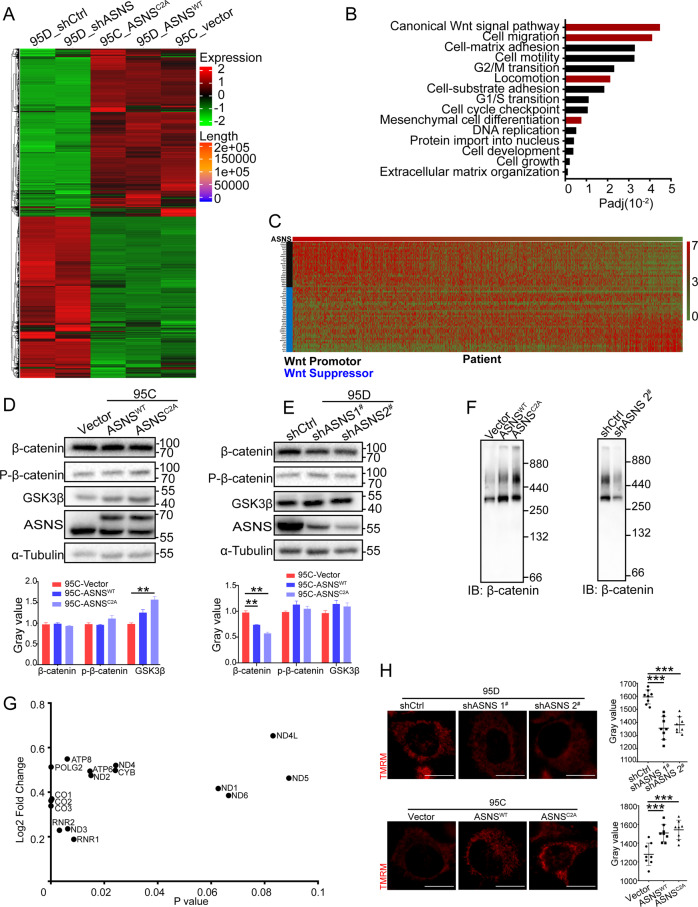
ASNS stabilizes the β-catenin complex and upregulates mitochondrial potential. The whole-cell RNA was extracted and put into RNA sequencing (**A**). Some pathways were enriched in the differential gene expression pool (**B**). **C** RNA sequencing data was collected from the TCGA, then ranked Wnt pathway-related genes by gradient ASNS mRNA level from higher to lower. β-catenin, an important secondary messenger was tested in ASNS overexpressed (**D**) and knockdown (**E**) cell lines. **F** The stability of β-catenin complexes was detected by blue native page in overexpressed and knockdown cell lines. **G** 11 mitochondrial coding mRNAs and POLG2 mRNA were found in differential expression gene pools. **H** TMRM was a special probe to detect mitochondrial potential. The mitochondrial potential was detected in overexpressed and knockdown cell lines; the white bar was 20 μm. Error bars were expressed as mean ± S.D. *P* < 0.05 is significant statistic difference, *p < 0.05, **p < 0.01, ***p < 0.001. Representative images from three independent experiments are shown.

For investigating why ASNS promoted cell invasiveness without strictly requiring endogenous asparagine synthesis, whole-cell RNA was extracted for RNA sequencing (Fig. 3A). In the differential gene expression pool, many genes were enriched in the canonical Wnt signal pathway (Fig. 3B), which was a conserved pathway to promote cell metastasis, survival, and proliferation. Furthermore, a lung-cancer RNA sequencing data were collected from the TCGA database (https://xenabrowser.net/datapages/). Wnt pathway-related genes were ranked by gradient ASNS mRNA level from higher to lower, higher ASNS mRNA level was positively correlated with the level of Wnt upregulated genes and negatively related with the level of Wnt downregulated genes (Fig. 3C). Then whole-cell proteins were extracted for SDS page assay, knockdown ASNS decreased β-catenin level (Fig. 3E), but overexpressed ASNS just had a weak effect on β-catenin expression (Fig. 3D). Then we extracted native β-catenin complex into blue native page assay, both ASNS^WT^ and ASNS^C2A^ upregulated β-catenin complex stability (Fig. 3F), especially in ASNS^C2A^ type, we found more stable β-catenin supercomplexes existed between 440 kD and 880 kD. Oppositely, knockdown ASNS decreased β-catenin complex level (Fig. 3F).

**Fig. 4:**
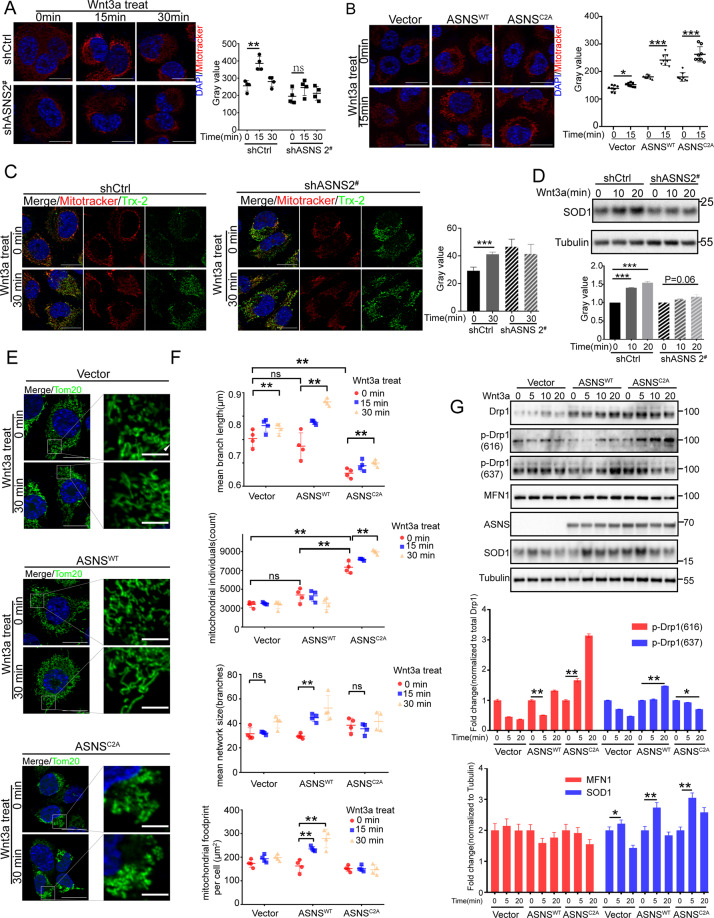
ASNS modulates mitochondrial response to wnt3a stimuli. Mitotracker Red was a special probe to detect mitochondrial potential. Under wnt3a treated, mitochondrial potential was detected in ASNS knockdown (**A**) and overexpressed (**B**) cell lines, mitotracker values were read by ImageJ and analyzed by Student’s *t* test, the white bar was 20 μm. **C** Thioredoxin 2 (Trx-2) was an important redox protein in mitochondrial, the length of the bar was 10 μm. **D** superoxide dismutase 1 (SOD1) played a key role in cellular redox reactions, and whole-cell proteins were extracted for detection. **E** under wnt3a treated, mitochondrial shapes were shown by Tom20 staining, which was an outer membrane protein of mitochondria, the bar was 20 μm. **F** We used Fiji/MiNA (tools for mitochondrial morphology research) to quantify mitochondrial morphology in (**E**), the difference of groups was analyzed by Student’s *t* test. **G** Mitochondrial morphology associated proteins were detected by western blot, The p-Drp1 (616) was a marker of mitochondria-fission promoter, the p-Drp1 (637) was a marker of mitochondria-fission suppressor, the different protein levels were detected by ImageJ and analyzed by Student’s *t* test. Error bars were expressed as mean ± S.D. *P* < 0.05 is significant statistic difference, *p < 0.05, **p < 0.01, ***p < 0.001. Representative images from three independent experiments are shown.

ASNS was an essential enzyme for asparagine products, asparagine was a key metabolite in the cellular response to mitochondrial dysfunction and coupled mitochondrial respiration for tumor growth [11, 12]. In whole-cell RNA sequencing data, overexpressed ASNS affected a lot of mitochondria-coding mRNA levels, such as MT-COI, MT-COII, and MT-COIII, these were core components of complex IV (Fig. 3G). Knockdown ASNS decreased mitochondrial potential, oppositely overexpressed ASNS^WT^ or ASNS^C2A^ could increase mitochondrial potential (Fig. 3H). The wnt3a was an effective activator to stimulate Wnt signal transduction in mammal cells [13–15]. Less response to wnt3a by knockdown ASNS caused slight increase of mitochondrial potential and redox response compared to wild-type cells (Fig. 4A, C, D). Oppositely, overexpressed ASNS^WT^ or ASNS^C2A^ enhanced cellular response to wnt3a stimuli with higher mitochondrial potential and redox response than vector cells (Fig. 4B, G). Mitochondrial dynamics was another important function of mitochondria. Wnt3a obviously promoted mitochondrial fusion In ASNS^WT^ cells compared to Vector or ASNS^C2A^ cells (Fig. 4E). The ImageJ/MiNA (tools for mitochondrial morphology research) was used for quantified mitochondrial morphology [16]. At baseline, mean branch length was shorter and mitochondrial individuals were higher in the ASNS^C2A^ group than ASNS^WT^ or Vector group, suggesting that more mitochondrial fragments existed in ASNS^C2A^ cells (Fig. 4F). Under wnt3a treated, mean branch length, mean network size, and mitochondrial footprint increased obviously in ASNS^WT^ type, which indicated that ASNS^WT^ elevated response to wnt3a with mitochondrial fusion increased (Fig. 4F). Mechanically, in ASNS^WT^ group, phosphorylated DRP1(Ser616) was decreased at 5 min after wnt3a treated, phosphorylated DRP1(Ser637) was increased at 20 min after wnt3a treated (Fig. 4G). The reversible phosphorylation of DRP1 at Ser637 and Ser616 was the main cause of mitochondrial fission [17, 18], so the higher level of phosphorylated DRP1(Ser637) and lower phosphorylated DRP1(Ser616) were responsible for increased mitochondrial fusion under wnt3a stimuli in ASNS^WT^ group. When wnt3a treated, phosphorylated DRP1(Ser 616) increased and phosphorylated DRP1(Ser 637) decreased in ASNS^C2A^ cells (Fig. 4G).

### ASNS promotes GSK3β phosphorylation and stabilizes the β-catenin complex

**Fig. 5:**
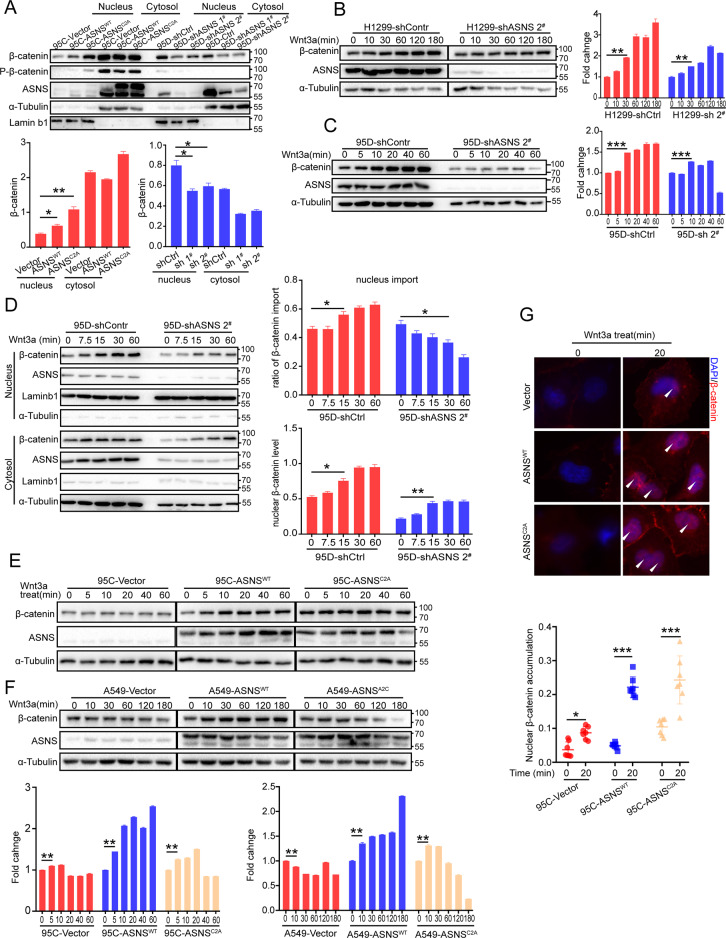
ASNS promotes β-catenin accumulation and translocation into nucleus. **A** Cell components of cytosol and nucleus were segregated by the Nuclear and Cytoplasmic Extraction Reagents. Lamin b1, a marker of nuclear component, α-tubulin, a marker of cytosolic component; levels of nuclear *β*-catenin were normalized to Laminb1, and levels of cytosolic *β*-catenin were normalized to Tubulin. Wnt 3a was a special activator for Wnt pathway and was always used to drive Wnt cascade in vitro. The whole-cell β-catenin accumulation was detected by western blot when wnt3a treated in cell lines of H1299 (**B**), 95D (**C**), 95C (**E**), and A549 (**F**). All levels of *β*-catenin in gradient time after wnt3a treatment were normalized to initial levels of *β*-catenin (0 min). Furthermore, the translocation of β-catenin into nucleus was detected by western blot (**D**) and immunofluorescence (**G**). The different protein levels between groups were tested by Student’s *t* test. Error bars were expressed as mean ± S.D. *P* < 0.05 is significant statistic difference, *p < 0.05, **p < 0.01, ***p < 0.001. Representative images from three independent experiments are shown.

β-catenin was an essential secondary messenger in the Wnt pathway, the increased nuclear β-catenin was a vital marker of Wnt pathway activity [19, 20]. We segregated cell components into cytosol and nucleus by Nuclear And Cytoplasmic Extraction Reagents [21]. Knockdown ASNS downregulated β-catenin level in nucleus, oppositely overexpressed ASNS^WT^ or ASNS^C2A^ upregulated β-catenin level in nucleus (Fig. 5A). Then, We detected whole-cell β-catenin accumulation under gradient time of wnt3a treatment, knocked down ASNS delayed increase or suppressed accumulation of β-catenin in 95D and H1299 cell lines (Fig. 5B, C). More important, knockdown ASNS decreased the imported ratio of β-catenin into nucleus and downregulated β-catenin level in nucleus when wnt3a treated (Fig. 5D). Nevertheless, overexpressed ASNS^WT^ or ASNS^C2A^ upregulated whole-cell β-catenin accumulation in 95C and A549 cell lines (Fig. 5E, F). Higher ratio of nuclear β-catenin import was detected in ASNS^WT^ or ASNS^C2A^ groups compared to Vector group under wnt3a treated (Fig. 5G).

**Fig. 6:**
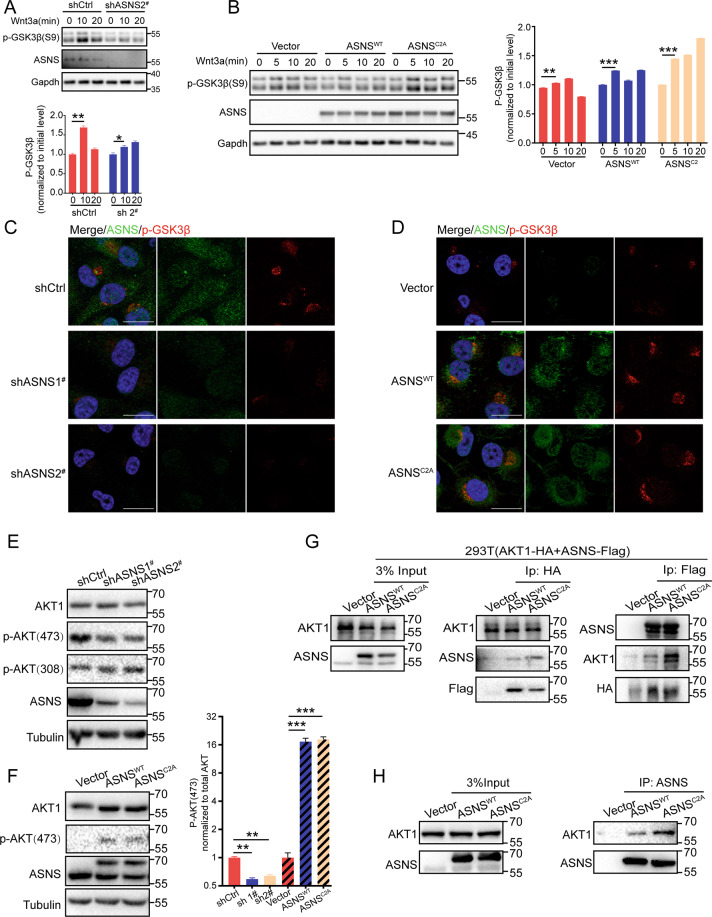
ASNS interacts with AKT and promotes GSK3β phosphorylation on ser9. p-GSK3β(S9) was an inactivated type of GSK3β, under Wnt3a treatment, the whole-cell phosphorylated GSK3β level was extracted by 2% SDS lysis buffer and detected by western blot in ASNS knockdown (**A**) and overexpressed (**B**) cell lines. **C**, **D** cells were permeabilized by 0.1% Triton X100 for 10 min, then phosphorylated GSK3β levels were detected by immunofluorescence. AKT was an essential upstream protein for GSK3β phosphorylation. Total AKT and phosphorylated AKT (active) levels were detected in ASNS knocked down (**E**) and overexpressed (**F**) cell lines. **G** AKT-HA and ASNS-FLAG plasmids were transfected into HEK-293T cell lines for 2 days, then purified AKT or ASNS proteins by magnetic beads for co-immunoprecipitated detection. **H** ASNS proteins were purified by IgG magnetic beads, then targeted for co-immunoprecipitated detection. The different protein levels between groups were tested by Student’s *t* test. Error bars were expressed as mean ± S.D. *P* < 0.05 is significant statistic difference, *p < 0.05, **p < 0.01, ***p < 0.001. Representative images from three independent experiments are shown.

Then we tried to find potential regulation of ASNS in the Wnt pathway, but there had no strong interactive proteins were found, like LRP6, GSK3β, β-catenin, or AXIN2 (data not shown). Knockdown ASNS decreased phosphorylation of GSK3β on Ser9 at baseline level (Fig. 6C) or under wnt3a treated (Fig. 6A). Oppositely overexpressed ASNS could increase phosphorylation of GSK3β at baseline (Fig. 6D) or under wnt3a treated (Fig. 6B). The phosphorylation of GSK3β on Ser9 is critical to stabilizing the β-catenin complex [19, 20, 22]. So, we focused on GSK3β and its upstream proteins. AKT was the main course of GSK3β phosphorylation. Phosphorylated AKT (Ser 473) was the active type to phosphorylate GSK3β on Ser9 [23, 24]. ASNS could interact with AKT (Fig. 6G, H). Knockdown ASNS suppressed AKT phosphorylation on Ser473 (Fig. 6E), overexpressed ASNS^WT^ or ASNS^C2A^ promoted AKT phosphorylation on Ser473 (Fig. 6F), suggesting that ASNS promoted phosphorylation of GSK3β on ser9 through activating AKT.

## Discussion

Tumor cells frequently require some metabolic changes to enhance their growth and metastasis [3, 11, 25]. Recent studies demonstrated that asparagine drove tumor growth and metastasis by modulating their survival, growth, and EMT pathways [5, 26]. Human asparagine synthetase is a key enzyme that catalyzes endogenous asparagine products [10]. In the mouse breast cancer model, high ASNS expression is positively correlated with the amount of circulating tumor cells and invasive potential [5]. ASNS overexpression can rescue cell apoptosis and cycle arrest owing to ATF4 deficiency or nutrition stress [8, 9]. But some phenotypes in primary tumors and ASNS-induced metastasis have so far not been explained.

The promotion of ASNS in tumor metastasis was confirmed in our study. The individuals with higher ASNS had poorer prognoses and more risk of metastasis than those with lower ASNS. Under asparagine starved, impaired invasiveness could be rescued by overexpressing ASNS^WT^ or ASNS^C2A^, suggesting that ASNS promoted cell invasiveness by alternative pathways. Mechanically the Wnt signal pathway and mitochondrial response were responsible to ASNS induce metastasis.

The Wnt/β-catenin pathway was conserved and widespread in tumor growth and metastasis. In the absence of Wnt ligands, β-catenin was packaged with a destruction complex containing scaffold proteins: GSK3β, AXIN, APC, CK1. Without stimuli, β-catenin was phosphorylated by GSK3β, ubiquitinated by β-TrCP, and targeted for proteasomal degradation [19, 20, 22]. In our study, ASNS could promote GSK3β phosphorylation on Ser9, which was an inactive type of GSK3β, to stabilize the β-catenin complex. Especially in the ASNS^C2A^ type, GSK3β phosphorylation was more apparent, as result, more β-catenin could avoid proteasome-dependent degradation and translocate into nucleus when the Wnt pathway was activated. The ASNS^C2A^ induced stability of the β-catenin complex would explain why ASNS^C2A^ could rescue invasive impairment by endogenous asparagine decrease. In Wnt pathway, we just found some weak interactions with ASNS, like LRP6 and GSK3β (data not shown). These could not explain how ASNS took part in the modulation of Wnt pathway. What is more, GSK3β phosphorylation (ser9) was noticeable when ASNS was overexpressed, and GSK3β phosphorylation was vital to hold Wnt pathway cascade. AKT was the main cause of GSK3β phosphorylation [27]. Full activation of AKT was through phosphorylation of Thr308 and Ser473 of AKT, and active AKT inactivated GSK3β by phosphorylating its amino-terminal regulatory domains on Ser9 [27]. ASNS could interact with AKT and upregulate the phosphorylation of AKT on Ser473, then active AKT phosphorylated GSK3β on Ser9, suggesting that ASNS modulated Wnt pathway through AKT/GSK3β/β-catenin axis.

ASNS and mitochondrial function were also intertwined. Mitochondria-derived aspartate was the source of asparagine product, and dietary asparagine was a limitation for cell proliferation with respiration impaired [12]. Overexpressed ASNS^WT^ or ASNS^C2A^ could help cells to maintain high mitochondrial potential, the changes in mitochondrial respiratory chains could partially explain how ASNS influenced mitochondrial potential. Under wnt3a treated, ASNS^WT^ or ASNS^C2A^ elevated mitochondrial response to wnt3a stimuli with mitochondrial potential increased, suggesting ASNS was an important modulator in process of mitochondrial response to wnt3a stimuli. We use ImageJ/MiNA (tools for mitochondrial morphology research) to analyze mitochondrial morphology in immunofluorescent images [16]. At baseline, more mitochondrial fragments existed in ASNS^C2A^ cells. When wnt3a treated, ASNS^WT^ elevated response to wnt3a with mitochondrial fusion increased. Mechanically, In ASNS^WT^ type, wnt3a stimuli could enhance mitochondrial fusion because of increased phosphorylation of DRP1 on Ser637 and decreased phosphorylation of DRP1 on Ser616. However, in ASNS^C2A^ type, wnt3a upregulated phosphorylated DRP1(Ser 616) and downregulated phosphorylated DRP1(Ser 637), mitochondrial fragments were increased under wnt3a treatment, which indicated that ASNS^C2A^ responded to wnt3a stimuli with mitochondrial fission increased. Zhao et al. reported that mitochondrial fission upregulated tumor metastasis, silencing Drp1 inhibited lamellipodia formation, a key step for cancer metastasis, as well as suppressed recruitment of mitochondria to lamellipodial regions [28]. ASNS^C2A^ upregulated mitochondrial fission, as well as maintained high mitochondrial potential to promote cell invasiveness.

Asparagine product was an important function of ASNS, which took part in protein synthesis and TCA cycle. As previously reported, ASNS was a vital effector of some oncogene pathways, like the PI3K-AKT-mTOR pathway [25] and the KRAS-ATF4 pathway [8]. Asparagine product was always considered as the main cause of ASNS-induced growth and metastasis. Limited asparagine by knockdown of ASNS reduced the number of circulating tumor cells without affecting primary tumor growth [5]. In our study, we found that ASNS promoted cell growth and invasiveness depending on differential pathways, asparagine product was more critical for ASNS to enhance cell growth. However, ASNS promoted invasiveness and relied on the activity of Wnt pathway cascade and mitochondrial functions without requiring endogenous asparagine products. After knockdown of ASNS, the growth of primary tumor could be supported by extracellular asparagine uptaking, but cell invasiveness was impaired by double aspects: limited asparagine and asparagine-independent dysfunctions. In our study, we focused on the asparagine-independent functions of ASNS. The double effect of ASNS should be taken into consideration when we suffer some questions, like asparagine availability, ASNS-related cell dysfunction, metastasis or growth, even for combination therapy of anti-cancer.

## Materials and methods

### Cell lines and culture

The human giant cell lung-cancer cell lines 95C (low metastasis) and 95D (high metastasis) were validated by Chun et al. [29] and gifted from the National Collection of Authenticate Cell Culture. 95C and 95D were cultured in RPMI 1640 medium supplemented with 10 % fetal bovine serum (FBS) and 1 %penicillin-streptomycin (Thermo Fisher Scientific). A549, H1299, and 293T were provided as gifts from the National Collection of Authenticate Cell Culture and cultured in DMEM supplemented with 10 % fetal bovine serum and 1 %penicillin-streptomycin. The Wnt3a cell line was gifted from Deng et al. [13] and cultured in DMEM medium. The mycoplasma contamination detection had done in all cell lines. In asparagine starved assays, 95C and 95D were cultured in a low asparagine medium.

### Human NSCLC samples collection

NSCLC surgical samples were collected from Xiangya hospital. The matched para-tumor tissues were taken at least 5 mm away from the margin of the tumor. Human surgical samples were immediately frozen in liquid nitrogen and transferred to −80 °C refrigerators. All samples were paraffin embedding and fixed in glass slides with formalin for immunohistochemistry analysis. This study was approved by the Institutional Review Board (IRB) of Xiangya Hospital. Written informed content was obtained from every patient. The study was conducted in accordance with the Declaration of Helsinki.

### Transwell assay

Plates with 8 μm-pore size chamber inserts (Corning, USA) were used for transwell assay. The chamber was coated with Matrigel, then seeded 2 × 10^4^ cells into the upper chamber. Cells were suspended in 200 μl RPMI 1640 medium with 0.1 % FBS in the upper chamber. In the lower chamber, 800 μl of RPMI 1640 medium was supplemented with 10 % FBS. After incubating at 37 °C for 24–48 h, some cells would migrate and invade through the Matrigel-coated membrane, attaching to the lower side of the membrane. Cells were fixed with 4 % paraformaldehyde for 10 min and stained with 0.1 % crystal violet for 25 min. Then, striking off upper cells that attached to the membrane’s inner side, invasive cells were imaged by CKX41 inverted microscope (Olympus, Japan) and counted by image J (ImageJ, RRID: SCR_003070).

### Western blotting

Prepared cells were resuspended with 200 μL of 2 % SDS cell lysis buffer (40 mM Tris/HCl pH7.4, 100 mM NaCl, 20 % Glycerol, 0.2 mM EDTA, 2 % SDS) on ice (Proteinase Inhibitor and PMSF were added freshly), then pipetted cell mixer until dissolved. Incubated cell lysis on the surface of ice for 5 min. Vortexed cell lysis again (max speed vortex), denaturalized protein in 95 °C, 10 min, then centrifuged under 13000 rpm, 15 min. The supernatants were collected for the next step. Western-loading samples were prepared by adding 4 × sample loading buffer (200 mM DDT and 4 mM PMSF were added) into samples. Loaded appropriated samples for electrophoresis, then transferred proteins from gel to PVDF membranes. After transfer, membranes were stained with 0.25 % Coomassie Blue R250 (Macklin, #B802269) for 30 s, then destained with 50 % ethanol (Sinopharm Chemical Reagent, # 10009218) until less background. Took photos by image device and removed Coomassie with methanol. Blocked with 5 % milk and incubated with primary antibody overnight at 4 °C. Then, washed with PBST (contained 0.1 % Tween20) for 5 min × 3 times. Incubated with the second antibody for 1 h at room temperature, slightly shaking. Then washed with PBST for 5 min × 3 times. And took photos by Chemiluminescence Gel Imaging System (ChampCheni910).

### Prepare samples for the Blue Native page and electrophoresis

Appropriate cells (5 cm dish) were collected on a cell bench and washed with ice-cold PBS. Discarded all supernatant and resuspended with cell lysis (1 % DDM, 20 mM Tris-HCl pH7.4, 0.1 mM EDTA, 50 mM NaCl, 10 % glycerol, 1 mM PMSF), pipetted 15 times then incubated on ice for 15 min, centrifuged under 13000 g, 15 min, collected supernatant and added 10 × loading dye (5 % Coomassie blue G250, 500 mM E-amino-n-caproic acid in 100 mM Bis-Tris pH7.0), slightly pipetted and centrifuged under 10000 g, 3 min, the supernatant samples were loaded into the gel. Before electrophoresis, gradient blue native gel was prepared (from 6 % to 16.5 %), then ran gel in Cathode buffer (15 mM Bis-Tris pH7.0, 50 mM Tricine, 0.02 % Coomassie Blue G250) and Anode buffer (50 mM Bis-Tris pH7.0) [30].

### Statical analysis

SPSS version 23 (IBM Corporation) and GraphPad Prism version 8.00 for Windows (GraphPad Software) were used for statistical analysis. The multivariate Cox regression analysis was used to evaluate prognosis‐related factors in the patient cohort. The Kaplan-Meier survival analysis (Log-rank (Mantel–Cox) test) was used to analyze patients’ survival. Unpaired t-test or ANOVA texted Between-group variations. *P* < .05 was considered to indicate statistical significance. The ImageJ/MiNA (tools for mitochondrial morphology research) was used for quantified mitochondrial morphology [16].

## Supplementary information

Supplemental Materials and Methods

reproducibility checklist

Original Data File

## Data Availability

The datasets generated and analyzed during the current study are available from the corresponding author on reasonable request.
