# Supplementary material for: Asparagine synthetase regulates lung-cancer metastasis by stabilizing the β-catenin complex and modulating mitochondrial response
Source: Cell Death Dis. 2022 Jun 23;13(6):566. doi: 10.1038/s41419-022-05015-0 (PMC9226154; doi:10.1038/s41419-022-05015-0)
Supplement: Supplementary file 1 — Supplemental Materials and Methods [file 41419_2022_5015_MOESM1_ESM.docx]

**Supplemental Materials and Methods**

*Cell lines and culture*

The human giant cell lung cancer cell lines 95C (low metastasis) and 95D (high metastasis) were validated by Chun et al. (1) and gifted from the National Collection of Authenticate Cell Culture. 95C and 95D were cultured in RPMI 1640 medium supplemented with 10% fetal bovine serum (FBS) and 1%penicillin-streptomycin (Thermo Fisher Scientific). A549, H1299 and 293T were provided as gifts from the National Collection of Authenticate Cell Culture and cultured in DMEM supplemented with 10 % fetal bovine serum and 1 %penicillin-streptomycin. The mycoplasma contamination detection had done in all cell lines. In asparagine starved assays, 95C and 95D were culture in low asparagine medium which contained(1L): 400 mg KCL, 3700 mg NaHCO_3_, 6060 mg NaCl, 200 mg MgSO_4_-7H_2_O, 264 mg CaCl_2_-2H_2_O, 1000 mg D-glucose, 15 mg Phenol red, 10 μl 10 mg/ml Fe (NO_3_)_3_, 40 ml 100 × MEM vitamin mix (Gibco, Catalog number: 11120052), 15 ml 1 M HEPES (Gibco, Catalog number: 15630106). Before cell culture, 20 % dialytic fetal bovine serum (Gibco, Catalog number: 30067185), 50 × MEM Amino Acids Solution (Gibco, Catalog number: 11130051) and 100 × L-Glutamine (Gibco, Catalog number: 25030081) were added.

*Preparation of Wnt3a Condition Medium and Cell Treatment*

The L-Wnt3a cells (L cells overexpression of Wnt3a) were gifted by Deng et al.(2). The cells were cultured in DMEM medium supplemented with 10 % (vol/vol) fetal bovine serum and G418. At confluence, the cells were split 1:10 and cultured without G418. Cell culture media were repeatedly collected every 3 days. The supernatants were filtered and diluted with serum-free DMEM (1:1). The Wnt signaling pathway was activated by using conditioned medium collected from L-Wnt3a cells.

*Human NSCLC samples collected*

NSCLC surgical samples were collected from Xiangya hospital. The matched para-tumor tissues were taken at least 5 mm away from the margin of the tumor. Human surgical samples were immediately frozen in liquid nitrogen and transferred to -80 ℃ refrigerators. All samples were paraffin embedding and fixed in glass slides with formalin for immunohistochemistry analysis. This study was approved by the Institutional Review Board (IRB) of Xiangya Hospital. Written informed content was obtained from every patient. The study was conducted in accordance with the Declaration of Helsinki.

*Transwell assay*

Plates with 8 μm-pore size chamber inserts (Corning, USA) were used for transwell assay. The chamber was coated with Matrigel, then seeded 2 × 10^4^ cells into the upper chamber. Cells were suspended in 200 μl RPMI 1640 medium with 0.1 % FBS in the upper chamber. In the lower chamber, 800 μl RPMI 1640 medium was supplemented with 10 % FBS. After incubating at 37 ℃ for 24-48 hours, some cells would migrate and invade through the membrane, attaching to the lower side of the membrane. Cells were fixed with 4 % paraformaldehyde for 10 min and stained with 0.1 % crystal violet for 25 minutes. Then, moved upper cells that attached to the membrane's inner side, at least 7 fields per group were imaged randomly and counted by CKX41 inverted microscope (Olympus, Japan) and image J (ImageJ, RRID: SCR_003070). The steps of the invasive-cell count were as follows: open the images with ImageJ, transfer the type into 8 bit and adjust the image’s threshold to find the cells. Then click “Process/fill holes” and “Process/watershed” to make cells more apparent. At last, click the button of “analyze particles”, the count and size of cells were measured.

*Scratch test*

Before scratching cells, three perpendicular lines were drawn on the backs of 6-well plates. 6 × 10^5^ cells that were under the logarithmic growth phase were plated in 6-well plates. After 24 hours, scratched three horizontal lines on the cell layer with a small tip, then washed the cell layer with PBS to discard suspended cells. Cell layers were cultured in a medium with 1 % FBS in 5 % CO_2_, at 37 °C. The scratch lines were photographed at 0, 12, 24, and 48 h by an inverted microscope (Olympus, Japan). Wound healing areas were measured by ImageJ, and the healing rate was calculated using the following formula: Wound healing rate = (initial scratch width - final healing width)/initial scratch width × 100%.

*Viral transduction and stable selection*

For lentivirus production in a 10 cm dish, 9 μg of helper plasmids (3 μg pMD2G and 6 μg psPAX2) and 9 μg of target plasmid (pLVX-ASNS-3Flag or pLKO.1-shASNS) were transfected into 293T cells by lipo2000 (Invitrogen, Catalog number: 11668030). Lipo2000 medium was replaced with 10 ml fresh DMEM after 6 h. Viral supernatants were collected after 48 h and centrifuged by 1500 rpm × 15 min. Target cells were infected with viral supernatants as well as 7 μg /mL polybrene (Sigma, St. Louis, MO) was added. 95D and H1299 were selected with 1 μg /mL puromycin for 8 days. 95C and A549 were selected by Flow Cytometer. All stable cell lines were identified by western blot.

*Total RNA extraction and Real-Time Polymerase Chain Reaction Analysis*

Total RNA was isolated from cell lines or paired tissues by the Total RNA Extractor kit (Sangon Biotech, catalog number: B511311). RNA was then confirmed by the visibility of 18S and 28S RNA bands under UV light. 1μg of total RNA was reversed to cDNA by Reverse Transcription kit (Takara, Catalog number: RR037A). In Real-Time PCR buffer mixer, paired primers were used for amplification as follows: ASNS_F, 5' -AGCACGAACTGTTGTAATGTCA-3', ASNS_R, 5’-GGAAGACAGCCCCGATTTACT-3’; GAPDH_F, GCACCGTCAAGGCTGAGAAC, GAPDH_R, TGGTGAAGACGCCAGTGGA. Amplification reactions were performed in 20 μl volume of the SYBR Green I mixture (Vazyme, Catalog number: Q711-02) as follows: 10 μl 2 × ChamQ Universal SYBR qPCR Master Mix, 0.4 μl primer mix, 0.1 μl template, and some RNAase free water up to 20 μl. All the reactions were performed in triplicate in an iCycler iQ system (Bio-Rad, Richmond, CA). The thermal cycling conditions were as follows: 95 °C for 3 min, 40 cycles of 95 ℃ for 15 s, and 60 °C for 60 s. All PCR products from each primer pair were subjected to a melting curve analysis.

*Western blotting*

Appropriate cells (5 cm dish) were collected and washed with ice-cold PBS. Discarded all supernatant and resuspended with 200 μl of 2 % SDS cell lysis buffer (40 mM Tris/HCl pH7.4, 100 mM NaCl, 20 % Glycerol, 0.2 mM EDTA, 2 % SDS) on ice (Proteinase Inhibitor and PMSF were added freshly). Pipetted cell mixer until cells dissolved. Incubated cell lysis on the surface of ice for 5 min. Vortexed cell lysis again (max speed vortex), denaturalized protein in 95 ℃, 10 min, then centrifuged under 13000 rpm, 15 min. The supernatants were collected for next step. BCA assays were used to detect protein concentration. All samples' concentrations were calculated and adjusted to the same concentration with cell lysis buffer. Western-loading samples were prepared by adding 4 × sample loading buffer (200 mM DDT and 4 mM PMSF were added) into the samples. Loaded appropriated samples for electrophoresis under 80 V for 30 min and 120 V for an appropriate position in gel, then transferred proteins from gel to PVDF membranes under 250 mA for 90 min. For high-weight proteins, 400 mA and 80 min were suitable. After transfer, membranes were stained with 0.25 % Coomassie Blue R250 (Macklin, #B802269) for 30 s, then washed with ddH_2_O once and 50 % ethanol (Sinopharm Chemical Reagent, # 10009218) for a few minutes until less background. Took photos by image device and removed Coomassie with methyl alcohol. Before reaction with primary antibody, 5% milk blocking buffer (in PBST, contained 0.1 % Tween20) was added to block membranes for one hour (slightly shaker). Incubated with appropriately diluted primary antibody overnight at 4 ℃. Primary antibody: ASNS (1:1000, Santa Cruz Biotechnology Cat# sc-365809, RRID: AB_10843357), β-catenin (1:1000, Cell Signaling Technology Cat# 8480, RRID: AB_11127855), active β-catenin (1:500, Cell Signaling Technology Cat# 4270, RRID: AB_1903918), GSK3β (1:2000, Proteintech Cat# 22104-1-AP, RRID: AB_2878997), P-GSK3β (1:1000, Ser9, Cell Signaling Technology Cat#5558), AKT1 (1:1000, Cell Signaling Technology Cat#2938, RRID: AB_915788), P-AKT (1:500, Ser473, Cell Signaling Technology Cat#4060), P-AKT (1:500, Ser308, Cell Signaling Technology Cat# 13038), Tubulin (1:5000, Santa Cruz Biotechnology Cat# sc-8035, RRID: AB_628408), DRP1(1:1000, Cell Signaling Technology Cat# 8570, RRID: AB_10950498), P-DRP1(1:500, Ser616, Cell Signaling Technology Cat#3455, RRID: AB_2085352), P-DRP1(1:500, Ser637, Cell Signaling Technology Cat# 4867). Then, washed with PBST for 5 min × 3 times. Incubated with the second antibody for 1 h at room temperature, slightly shaking. Second antibody: Peroxidase-AffiniPure Goat Anti-Mouse IgG (H+L) antibody (1:5000, Jackson ImmunoResearch Labs Cat# 115-035-146, RRID: AB_2307392) and Peroxidase-AffiniPure Goat Anti-Rabbit IgG antibody (1:5000, Jackson ImmunoResearch Labs Cat# 111-035-144, RRID: AB_2307391). Then, the PVDF membranes were washed with PBST 5 min × 3 times. Before taking photos by Chemiluminescence Gel Imaging System (ChampCheni910), all membranes should be stocked in PBST. Nuclear and cytoplasmic extraction reagents (Thermo, RRID:78835) were used to segregate nuclear and cytosolic proteins. Western blot bands were quantified by ImageJ as follows: open initial western bands in ImageJ, convert image into 8-bit type, then subtract background (Rolling ball radius: 50 pixels), invert the image and measure every band with the same rectangle. Export data into Excel and analyzed it by GraphPad Prism version 8.00 for Windows (GraphPad Software). Before analysis, the background values of all bands must be subtracted.

*Prepare samples for the Blue Native page and electrophoresis*

Appropriate cells (5 cm dish) were collected on a cell bench and washed with ice-cold PBS. Discarding all supernatant and resuspended with cell lysis (1 % DDM, 20 mM Tris-HCl pH7.4, 0.1 mM EDTA, 50 mM NaCl, 10 % glycerol, 1 mM PMSF), pipetted 15 times then incubated on ice for 15 min, centrifuged under 13000g, 15 min. Collected supernatant and added 10 × loading dye (5 % Coomassie blue G250, 500 mM E-amino-n-caproic acid in 100 mM Bis-Tris pH7.0), slightly pipetted and centrifuged under 10000g, 3min. The supernatant samples were loaded into the gel. Before electrophoresis, gradient blue native gel was prepared (from 6% to 16.5%), then ran gel in Cathode buffer (15 mM Bis-Tris pH7.0, 50 mM Tricine, 0.02% Coomassie Blue G250) and Anode buffer (50 mM Bis-Tris pH7.0).

*Immunoprecipitation*

Adhesive Cells were digested and washed with ice-cold PBS once. Then cell proteins were released softly by IP lysis (20 mM Tris-Cl pH 7.4, 135 mM NaCl, 1.5 mM MgCl_2_, 1 mM EGTA, 10% glycerol, 1% Triton X-100). The whole lysis was centrifugated at 15000 rpm × 5 min to collect the upper supernatant. IgG beads (Sigma, Catalog number: 16-125) were washed with IP lysis 3 times, then incubated with primary antibody for 1 h. Then cell lysates were incubated with primary antibody and IgG beads for 4 h at 4°C. Washed products 4 times with pre-cold IP lysis buffer, then resuspended beads with 1 × loading buffer and boiled in 95 ℃ for 5 min. Upper supernatant was used for western blot analysis.

*Immunohistochemistry*

Collected lung cancer samples were sent to the pathology department of Xiangya hospital for paraffin embedding and tissue sectioning. 92 paired lung cancer tissues were fixed on the same glass slide. The tissue was dehydrated and incubated with ASNS primary antibody (1:150, Santa Cruz Biotechnology Cat# sc-365809, RRID: AB_10843357) overnight, then blocked endogenous peroxidase by 0.3 % H_2_O_2_ for 10 min and incubated with secondary antibody for 1 h. Then, the tissue was subjected to horseradish peroxidase until a noticeable color change was achieved. The tissue was then stained with hematoxylin, followed by dehydrated and mounted. The tissue slides were imaged using the light microscope (Prior, L22OPK1). Whole IHC images were scored in the Quantitative Pathology Imaging System (PerkinElmer).

*Immunofluorescence*

Double coverslips were placed per well in a 6-well culture plate. Washed slips with PBS 3 times (PBS should be placed at room temperature). Seed 1 × 10^6^ cells per well and culture overnight, then fixed with 4 % PFA for 15 min; Permeabilizated with 0.1 % Triton X-100, RT, 15 min; Blocked with 2 % BSA in 0.05 %PBST, RT, 1h; Diluted primary antibody (1:500) in 1 % BSA, 4 °C, overnight; 2nd Ab (1:1000) in 1 % BSA, RT, 1 h; Washed with 0.05 %PBST 5 min; Incubated slips with DAPI or Hoechst (dissolved in 0.05 % PBST, 1:1000), 10 min; Rewashed in 0.05 % PBST for 5 min. At last, mounted slides with fluorescence quenching resistant sealing tablets. For the same experiment, all images were captured under the same shooting condition, like intensity, and exposure. For a group, at least 7 fields were captured randomly. Then analyzed by ImageJ as follows: open images with ImageJ, select “ImageJ/Plugins/Biop Channel Tools”. First, choose “Channels LUT selection” to reset channels’ colors; second, choose “B&C selection” to adjust channels’ contrast; third, choose “Montage Options” to show different channels in the same frame. Save these parameters and apply them to folders where the original images are. In the folder of “saving_selectedBC”, we can get the same contrasted images to analyze. For whole gray value of images, choose the image and click “Measure”, For single cell, cell should be selected by “Polygon selections”, then click “Measure”. For all images, the background values must be subtracted.

*Crystal violet staining assay*

Cells were plated into a 6-well plate (1000 cells/well) and cultured in an FBS-free medium for 14 days. The cells were fixed with 4 % paraformaldehyde for 10 min, then stained with 0.3 % crystal violet for 5 min, and washed 3 times with fresh PBS. Whole-cell colonies of a well in a 6-well plate were photographed by a general camera and counted by image J. The steps of clonal count were as follows: open the pictures with ImageJ, transfer the type into 8 bit and adjust picture’s threshold to find the interesting elements, like cells. Then click “Process/fill holes” and “Process/watershed” to make cells more apparent. At last, click the button of “analyze particles”, the count and size of cells were measured.

*Colony formation assay*

The bottom layer gel (20 % FBS, 40 % 2 × RPMI-1640 medium (Basal Medium Eagle) and 0.5 % agar) was prepared and added to each well of the 24-well plate (400 μl per well). Then gel was curdled at 37 °C. The top layer gel (25 % FBS, 37.5 % 2 × RPMI-1640, 0.375 % agar, and 0.8% of 2 mM L-glutamine) was prepared and suspended with cells. Then, 400 μl of the mixture (containing 1000 cells) was added upon the bottom layer gel and cultured at 37 °C, 5 % CO_2_ for two weeks. At least 7 fields (10 ×) were selected randomly under an inverted light microscope. The clone formations were counted by ImageJ. The steps of clone analysis were similar to the method of “Crystal violet staining assay”

*Statical analysis*

SPSS version 23 (IBM Corporation) and GraphPad Prism version 8.00 for Windows (GraphPad Software) were used for statistical analysis. The Multivariate Cox regression analysis was used to evaluate prognosis‐related factors in the patient cohort. The Kaplan-Meier survival analysis (Log-rank (Mantel-Cox) test) was used to analyze patients’ survival. Unpaired t-tests or ANOVA texted Between-group variations. P < .05 was considered to indicate statistical significance. The ImageJ/MiNA (tools for mitochondrial morphology research) was used for quantified mitochondrial morphology(3).

1. Li CY, Tan L, Zhang GJ, Li P, Tong C, Fan J, et al. Transcriptional regulation of urokinase receptor in high- (95D) and low-metastatic (95C) human lung cancer cells. Acta biochimica et biophysica Sinica. 2004;36(6):405-11.

2. Deng YZ, Yao F, Li JJ, Mao ZF, Hu PT, Long LY, et al. RACK1 suppresses gastric tumorigenesis by stabilizing the β-catenin destruction complex. Gastroenterology. 2012;142(4):812-23.e15.

3. Valente AJ, Maddalena LA, Robb EL, Moradi F, Stuart JA. A simple ImageJ macro tool for analyzing mitochondrial network morphology in mammalian cell culture. Acta histochemica. 2017;119(3):315-26.
