# Supplementary material for: Asparagine synthetase regulates lung-cancer metastasis by stabilizing the β-catenin complex and modulating mitochondrial response
Source: Cell Death Dis. 2022 Jun 23;13(6):566. doi: 10.1038/s41419-022-05015-0 (PMC9226154; doi:10.1038/s41419-022-05015-0)

Figure1A

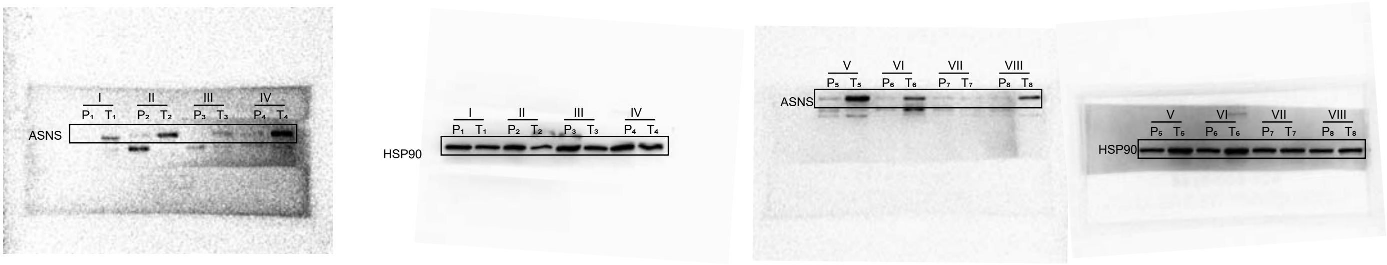

Figure2A

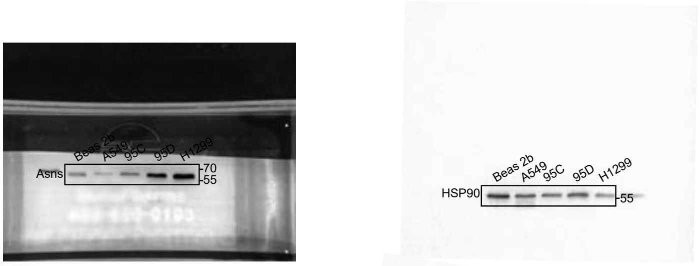

Figure2E

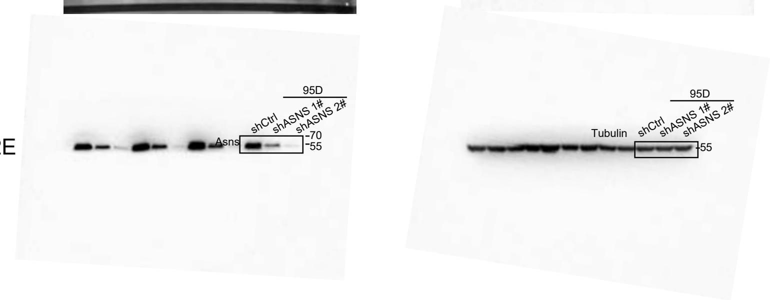

Figure2F

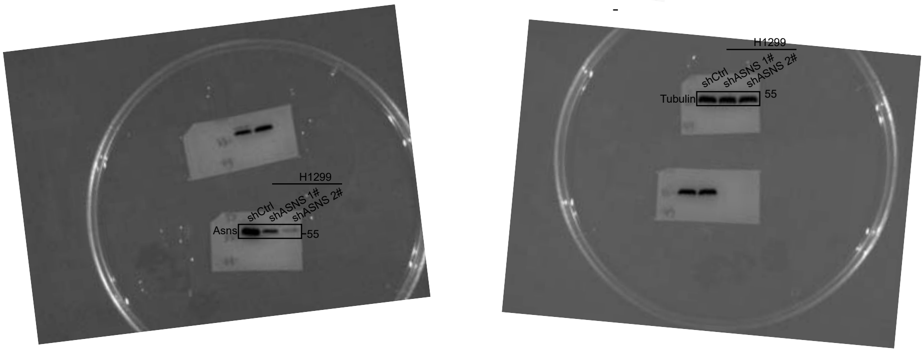

Figure2N

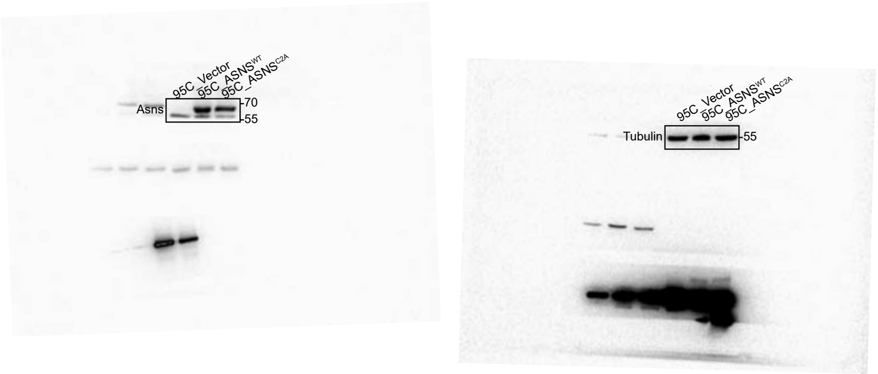

Figure 3D

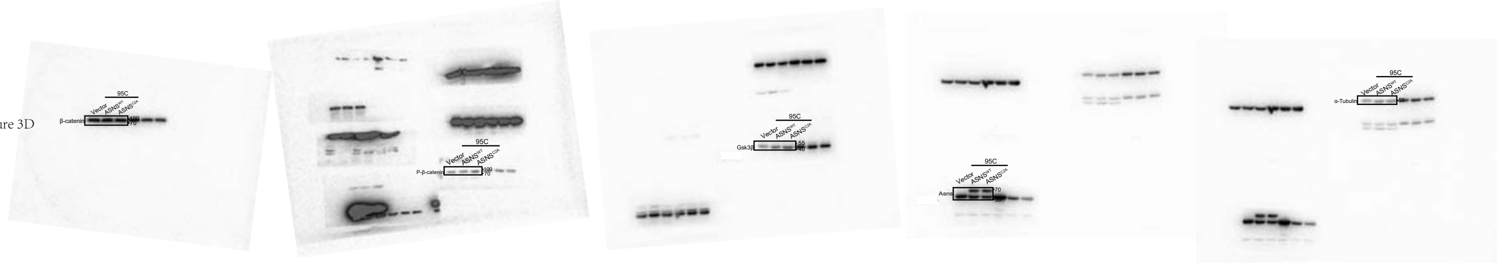

Figure 3E

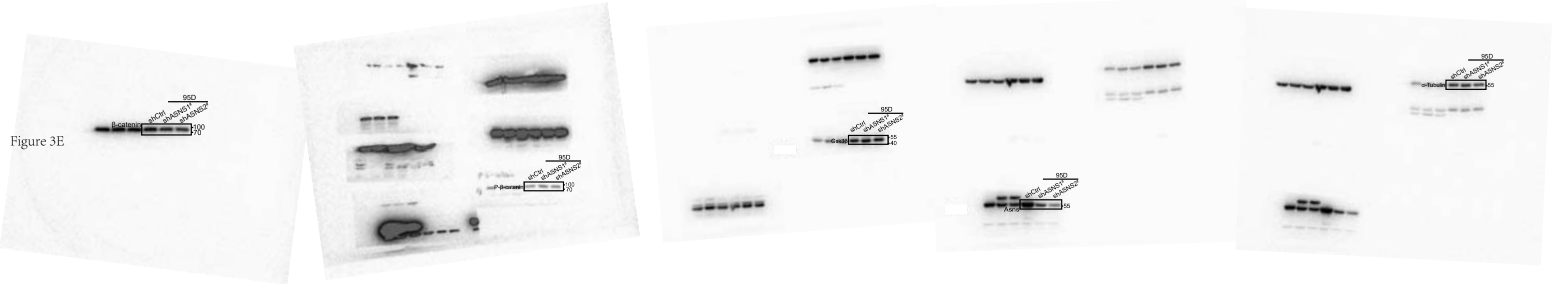

Figure 3F

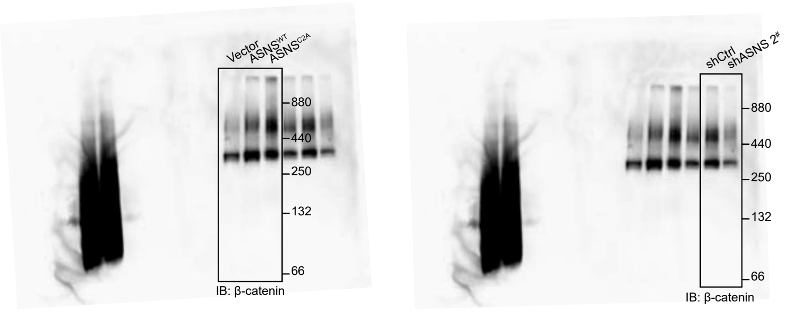

Figure 4D

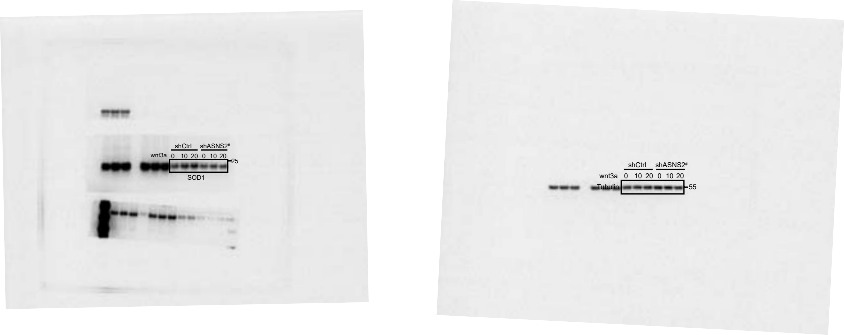

Figure 4G

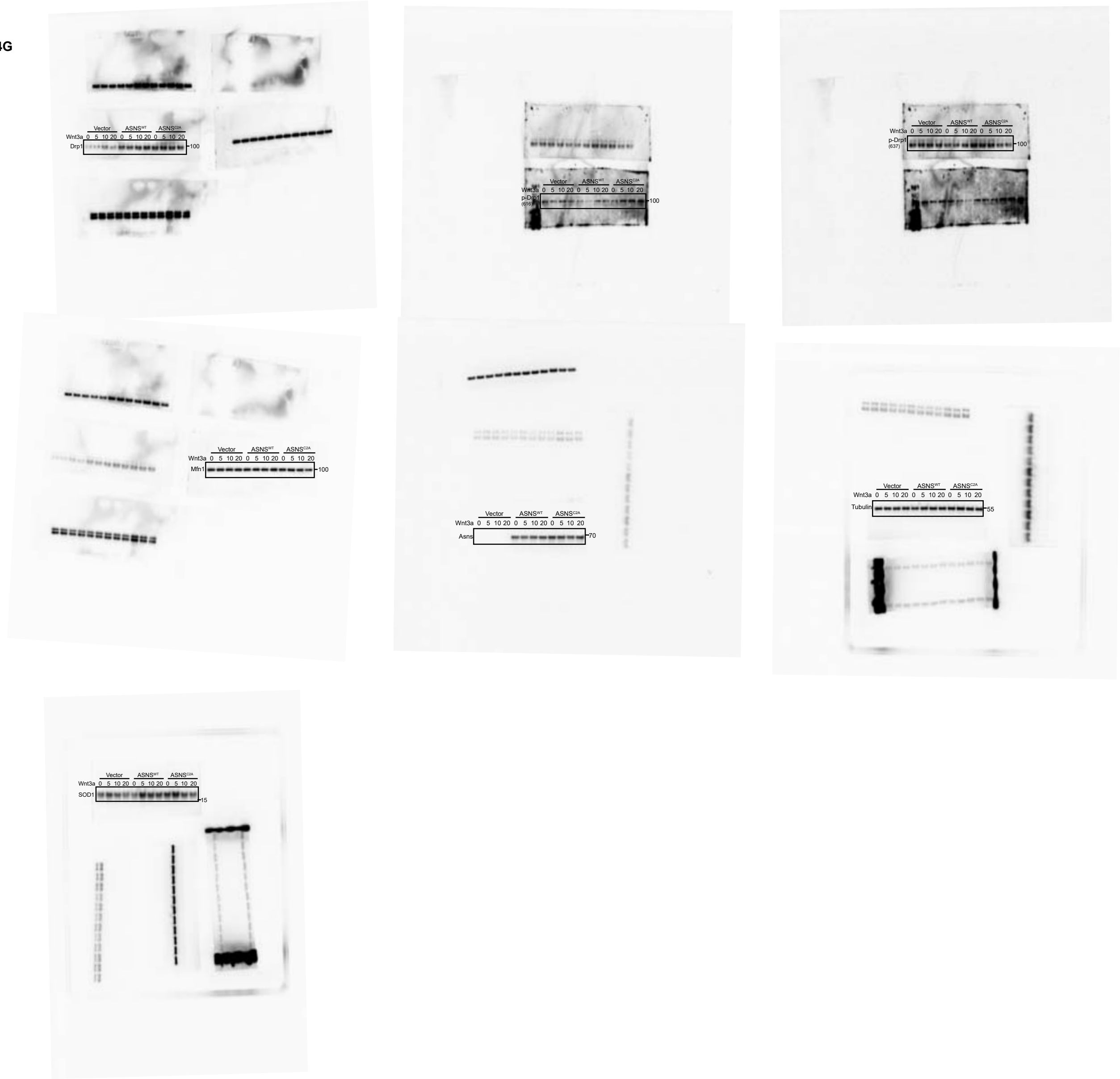

Figure 5A

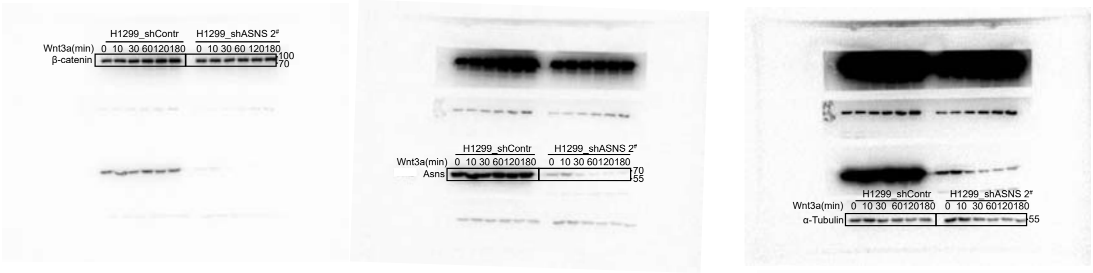

Figure 5B

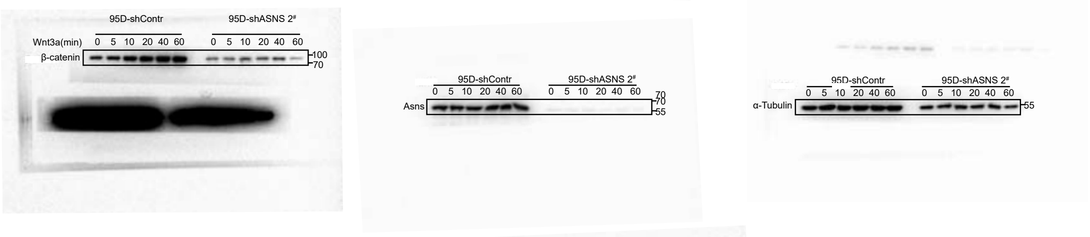

Figure 5C

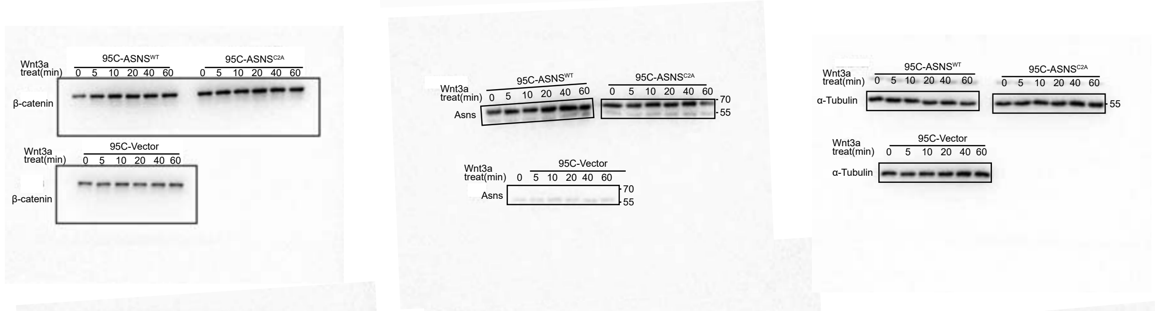

Figure 5D

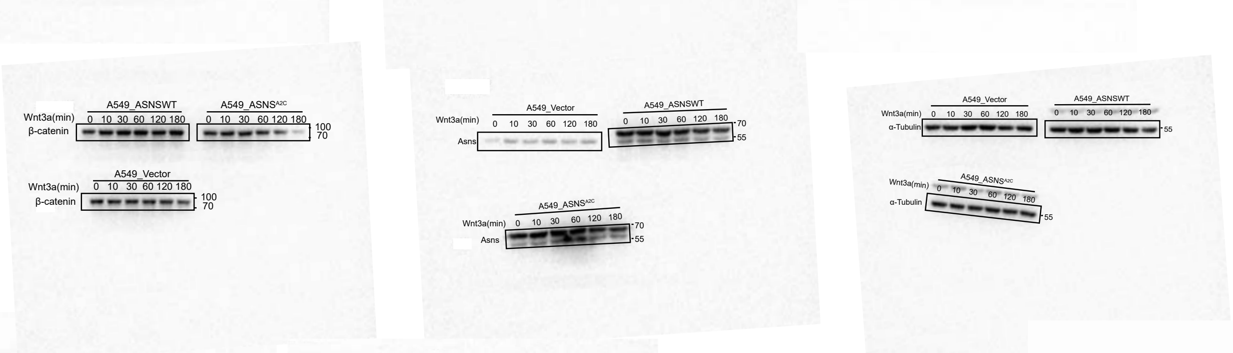

Figure 5F

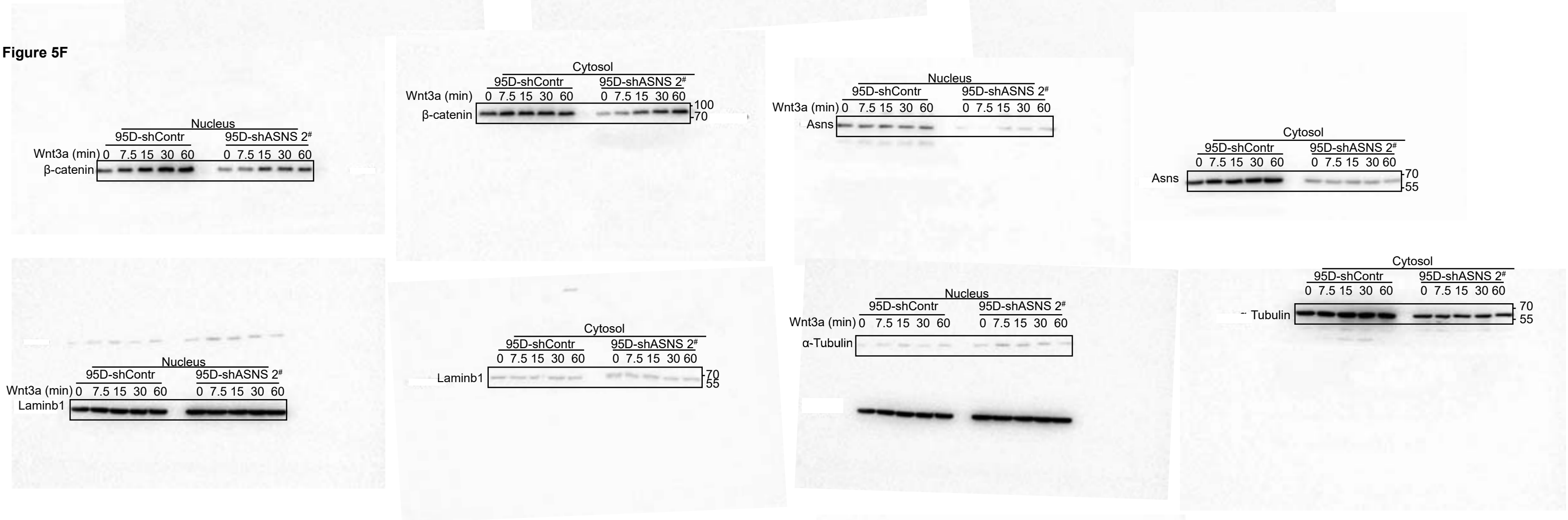

Figure 5G

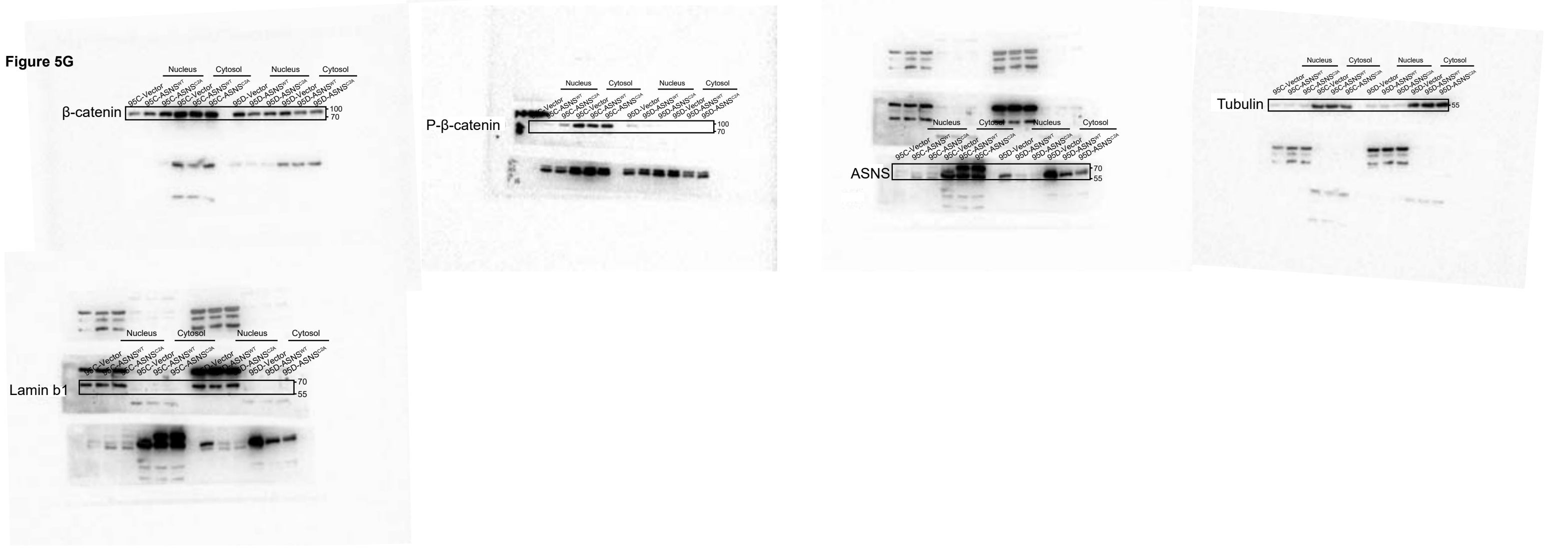

Figure 6A

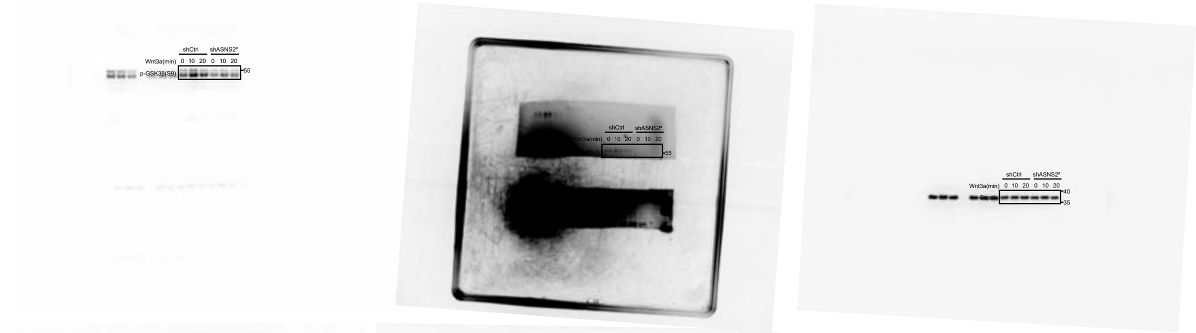

Figure 6B

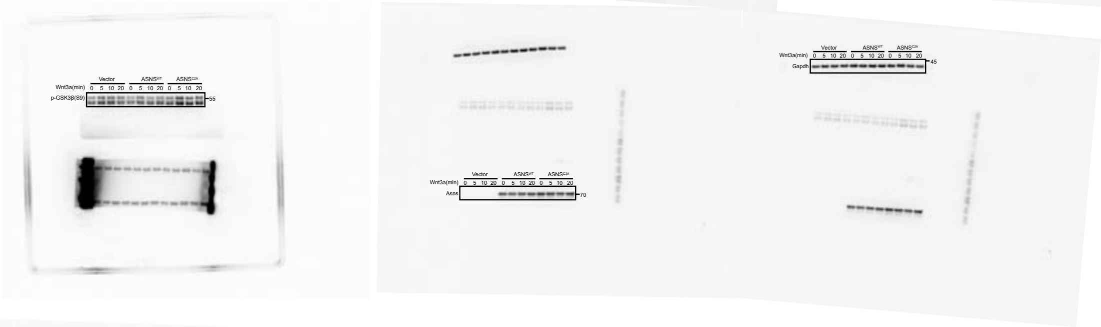

Figure 6E

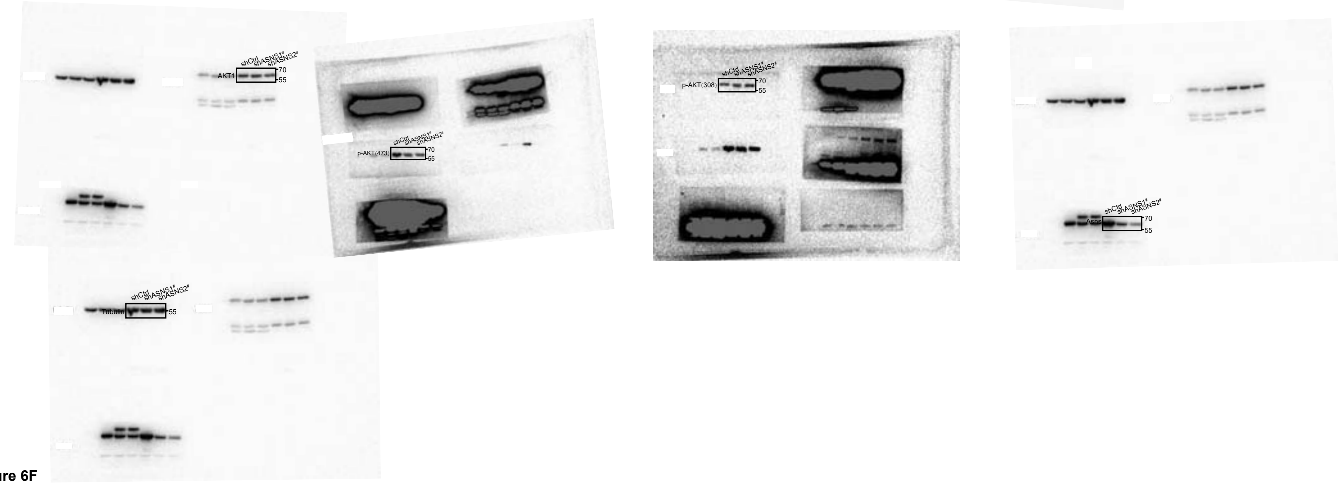

Figure 6F

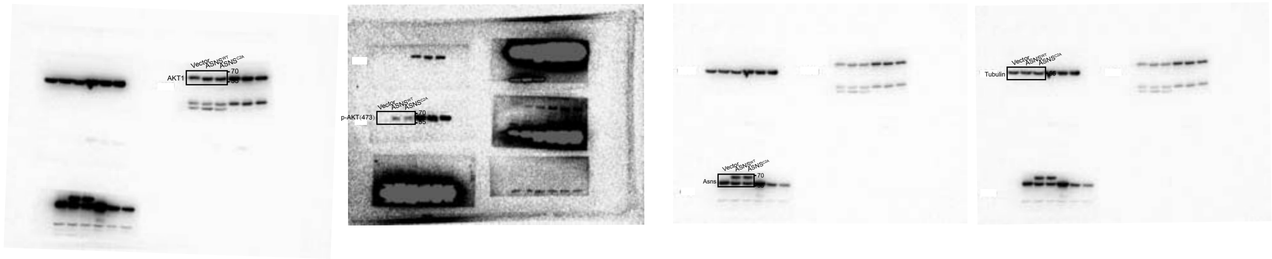

Figure 6G

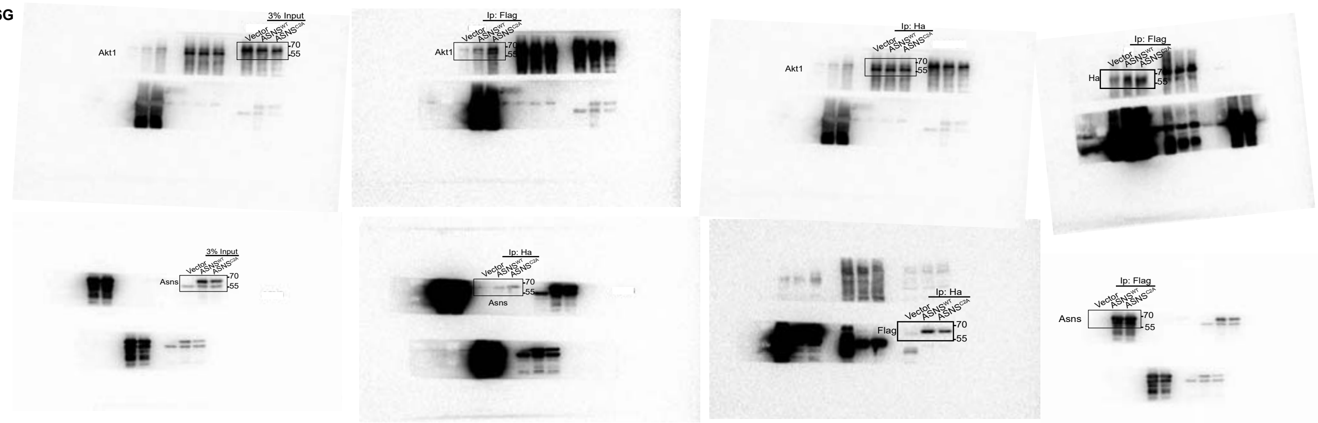

Figure 6H

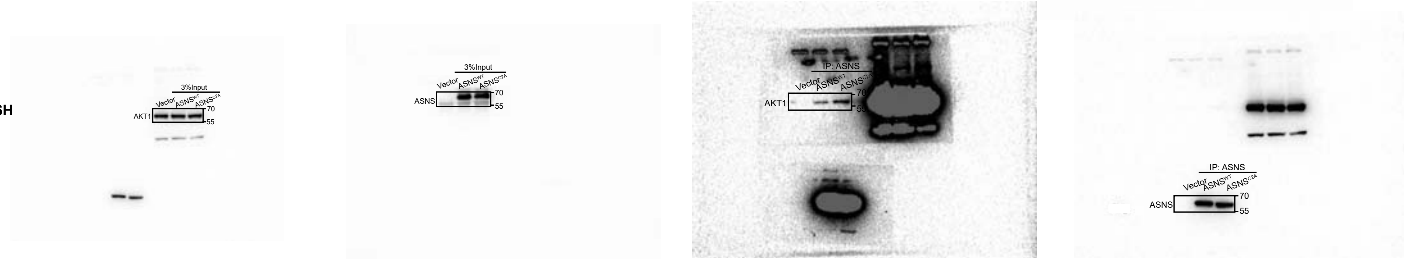

Supplement: Supplementary file 3 — Original Data File [file 41419_2022_5015_MOESM3_ESM.pdf]
